# The unfavorable clinical outcome of COVID-19 in smokers is mediated by H3K4me3, H3K9me3 and H3K27me3 histone marks

**DOI:** 10.2217/epi-2021-0476

**Published:** 2022-01-13

**Authors:** Milad Shirvaliloo

**Affiliations:** ^1^Infectious & Tropical Diseases Research Center, Tabriz University of Medical Sciences, Tabriz, Iran; ^2^Faculty of Medicine, Tabriz University of Medical Sciences, Tabriz, Iran

**Keywords:** COVID-19, epigenetic gene regulation, epigenetics and disease, H3K27me3, H3K4me3, HeK9me3, histone modifications, mdig, SARS-CoV-2, smoking

## Abstract

Smoking could predispose individuals to a more severe COVID-19 by upregulating a particular gene known as *mdig*, which is mediated through a number of well-known histone modifications. Smoking might regulate the transcription-activating H3K4me3 mark, along with the transcription-repressing H3K9me3 and H3K27me3 marks, in a way to favor SARS-CoV-2 entry by enhancing the expression of ACE2, NRP1 and NRP2, AT1R, CTSD and CTSL, PGE2 receptors 2–4, SLC6A20 and IL-6, all of which interact either directly or indirectly with important receptors, facilitating viral entry in COVID-19.

## Lay abstract

The role of smoking in development of several respiratory diseases has been clearly established. A significant proportion of these deleterious effects is mediated through epigenetic mechanisms, particularly histone modifications. Recent evidence indicates that smoking induces the expression of a mediator known as *mdig*, which in turn alters the transcription of several key proteins that have been implicated in development of COVID-19.

## The epigenetic link between smoking & COVID-19

Despite its negligible effects on the expression of ACE2 and TMPRSS2 in endothelial cells [1], cigarette smoking is still associated with increased risk of severe COVID-19. On the basis of the results of a 2021 meta-analysis on more than 800,000 COVID-19 patients, smoking predicts a 19% increased risk of death in patients with COVID-19 [2]. Strangely enough, this increased severity does not manifest in the form of gross pulmonary damage that is typically revealed by imaging techniques [3]. Although in the absence of overt gross pathology, it would be sensible to assume smoking-related chronic obstructive pulmonary disease (COPD) as the major culprit behind the worse clinical outcome of COVID-19 in smokers, rather than the adverse effects of smoking itself [1], one should not overlook the fact that less than one-third of smokers develop COPD [4]. Further, approximately 10% of nonsmoking individuals may show signs of impaired pulmonary function upon examination, indicating that pulmonary malfunction is not limited to smokers [5].

Known to regulate transcription without altering the DNA sequence, epigenetics is believed to be involved in the pathophysiology of COVID-19 in more than one way, but primarily through its expansive regulatory effects on the adaptive immune system and mechanisms of cell death by means of DNA methylation (DNAm) and histone modifications. DNA hypomethylation at three distinct CpG sites of *ACE2R –* namely, cg04013915, cg08559914 and cg03536816 – is suggested to confer an increased susceptibility to SARS-CoV-2 infection in human respiratory epithelial cells, which are the primary cell type affected throughout the course of COVID-19 [6]. Beside the influence of CpG sites on the altered expression of *ACE2R*, an investigation by Castro de Moura *et al.* on peripheral blood samples isolated from COVID-19 patients indicated a significant correlation among the clinical severity of COVID-19 and 44 CpG sites, a good majority of which occur at the proximity of sequences coding for inflammasome components, including *AIM2* and *HLA-C* [7]. As useful as they are for studying the DNAm signature of COVID-19, peripheral blood cells are themselves influenced by SARS-CoV-2-related dysregulation of DNAm, as confirmed by the frequent occurrence of lymphopenia in COVID-19 patients [8]. In addition to DNAm, the role of histone modifications, particularly transcription-repressing marks, has also been implicated in COVID-19, especially in the regulation of antiviral interferon response and transcription of interferon-stimulated genes such as *ACE2* and *IL6* [9], which are deregulated in patients with severe COVID-19.

Recent investigations have traced the footprint of epigenetics back to well before the onset of COVID-19 in a retrograde predisposing circuit, which is extensively influenced by external – or in a more proper sense, environmental – stimuli, such as air pollution and tobacco smoke [10] that are at the pinnacle of environmental issues in the modern world.

As biomedical scholars, it is professionally rewarding to know that epigenetic mechanisms alter the common transcriptional bridge between smoking and COVID-19 by trimethylation (me3) of particular lysine (K) residues at H3 and H4 histones [10] in the form of the heterochromatin-specific H3K9me3, H3K27me3 and H4K20me3 marks, as well as the euchromatin-specific H3K4me3 mark. Although the H3K9me3, H3K27me3 and H4K20me3 fall within the category of repressive histone marks – that is, they negatively regulate the expression of genes upon enrichment – H3K4me3 is an active histone mark that positively regulates transcriptional activity upon enrichment [11].

In the context of a presumed interplay between smoking and COVID-19, it is even more interesting when one realizes that all of these histone marks are somehow associated with *mdig* gene (GenBank: BE441202, https://www.ncbi.nlm.nih.gov/nuccore/BE441202) [10], a lung cancer-related, cell growth-regulating gene spanning ~30,000 base-pairs, located on chromosome 3 that was first identified in the alveolar macrophages of coal miners with a history of exposure to mineral dust. Mdig was essentially speculated to be a type of nuclear protein regulating transcriptional activity by means of chromatin remodeling because it contained several specific domains at its amino terminus known as JmjC domains, which are inherently associated with transcriptional regulation in bacterial and eukaryotic cells. Expressed normally in cardiac and skeletal muscle cells, mdig is undetectable in healthy pulmonary tissue. However, early investigations led predominantly by Zhang *et al.* suggested upregulation of *mdig* in the tumor tissue of patients with lung cancer [12]. The gene was later found to be upregulated in smokers in a pack-year-dependent manner, predicting poor overall survival in smokers diagnosed with lung cancer [13]. Given that *mdig* is generally a negative regulator of DNA and histone methylation, particularly the H3K9me3 mark [14], it was postulated that smoking, by means of downregulating DNAm and repressive histone marks through the induction of *mdig*, might lead to upregulation of several genes that predispose individuals to lung inflammation, fibrosis and malignancy [13]. For instance, *AHRR* is one such gene known to be hypomethylated at its cg05575921 CpG site, and thus overexpressed, upon chronic exposure to cigarette smoke, conferring an increased risk of lung cancer [15].

Silencing of *mdig* in human lung epithelial cells was recently shown to result in downregulation of several genes, which are overexpressed in the bronchoalveolar lavage fluid (BALF) of COVID-19 patients, indicating a potential positive regulatory effect for *mdig* on the genes involved in the pathogenesis of COVID-19 [10].

**Figure 1.**
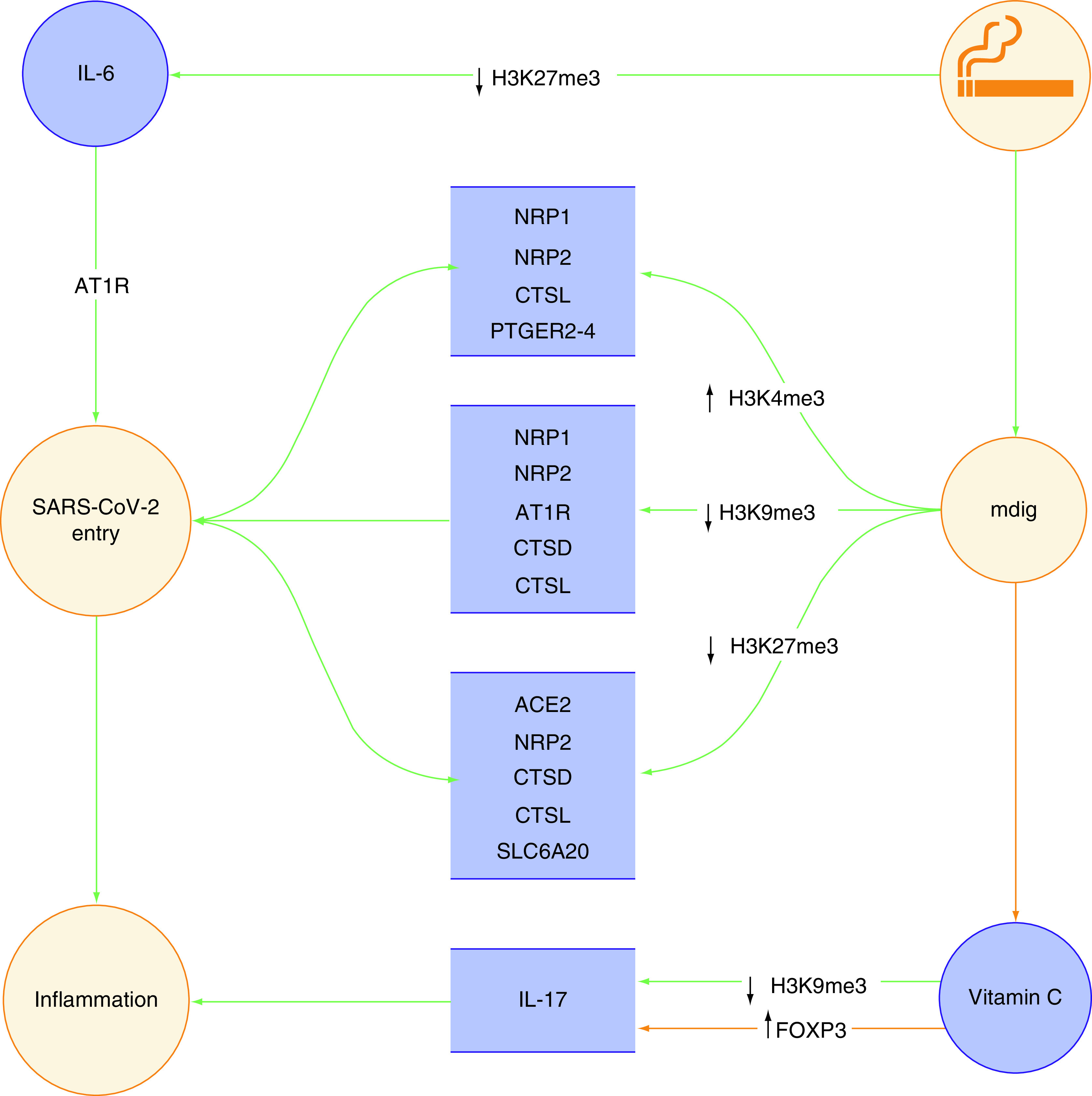
Epigenetic link between smoking and poor prognosis of COVID-19.

This perspective article attempts to provide the most detail-oriented picture regarding the epigenetically regulated bridge between smoking and COVID-19 in the hope of arriving at a better understanding of histone modifications and their impact on the disease-causing mechanisms of SARS-CoV-2. The transcriptional modifications discussed later in the article are illustrated in Figure 1.

## Smoking antagonizes repressive histone marks through upregulation of mdig

*Mdig* knockout results in significant enrichment of transcription-repressing histone modifications – namely, H3K9me3, H3K27me3 and H4K20me3 in human lung epithelial cells. In the case of H3K9me3, a near threefold enhancement was observed in *mdig*-deficient cells [10]. This appears to be of significant importance, particularly in COVID-19 patients because there is now evidence that H3K9me3 is downregulated at the promoters of certain pro-inflammatory genes driving macrophage-mediated inflammation [16]. The major reason for promoter-specific H3K9me3 diminution in COVID-19 patients is said to be the virus-induced inhibition of SETBD2, which catalyzes the trimethylation of H3 at lysine residue 9, i.e., H3K9me3 [16].

The genes downregulated as a result of H3K9me3 and H3K27me3 enrichment at their promoters are involved in glycan metabolism, which suggests a positive regulatory role for *mdig* in glycosylation through depletion of repressive histone marks. A post-translational modification of proteins with carbohydrate moieties, glycosylation is implicated in the viral entry of SARS-CoV-2 by affecting its S protein, which is discussed later in the article [10].

## Smoking triggers the cleavage of SARS-CoV-2 spike protein

The distinctive spike protein of SARS-CoV-2 is heavily involved in viral entry, which determines the onset of infection – and hence, COVID-19. The cleavage of the S protein is a prerequisite for the successful entry of SARS-CoV-2 into its host cell (e.g., lung epithelial cells). ACE2 and TMPRSS2 are two enzymes that mediate the recognition and cleavage of the S protein [17].

An oncogene induced by smoking, *mdig* is thought to accelerate SARS-CoV-2 entry because *mdig* knockout in human lung epithelial cells is accompanied by a lower cleavage rate of the SARS-CoV-2 spike protein. However, inhibition of the S protein cleavage upon *mdig* knockout does not seem to be caused by TMPRSS2 downregulation because there is no remarkable difference in the expression profile of TMPRSS2 between the *mdig*-deficient (*mdig*^-^) and *mdig*-expressing (*mdig*^+^) cells. Accordingly, there might also be other mediators involved in SARS-CoV-2 entry. Table 1 presents a detailed list of several epigenetically regulated *mdig*-induced candidate receptors/mediators that might contribute to SARS-CoV-2 entry.

**Table 1.** Epigenetically upregulated receptors/mediators associated with SARS-CoV-2 entry.

Receptor/mediator	Role in COVID-19	Histone marks in *mdig*^+^ epithelial cells	Ref.
ACE2	The main receptor mediating SARS-CoV-2 entry, the expression of ACE2 is maintained, if not upregulated, as a result of the arsenic-induced impaired activity of EZH2.	↓ H3K27me3	[10,18,19]
NRP1	Highly expressed in the respiratory tract epithelial cells, NRP1 bind the S1 segment of the SARS-CoV-2 spike protein following its cleavage by furin.	↑ H3K4me3 ↓ H3K9me3	[10,20]
NRP2	Similar to NRP1.	↑ H3K4me3 ↓ H3K9me3 ↓ H3K27me3	[10,21]
AT1R	Facilitates SARS-CoV-2 entry through receptor-mediated endocytosis of sACE2-S complex following the interaction of viral spike protein with soluble ACE2.	↓ H3K9me3	[10,22,23]
CTSD	Potentially facilitates SARS-CoV-2 entry through positive regulation of furin by means of osteopontin.	↓ H3K9me3 ↓ H3K27me3	[10,24–26]
CTSL	Elevated in the serum of COVID-19 patients, CTSL mediates viral entry by participating in the cleavage of the viral S protein.	↑ H3K4me3 ↓ H3K9me3 ↓ H3K27me3	[10,27]
PTGER2-4	Upregulation of PGE2 receptors might potentiate the positive regulatory effect of PGE2 on ACE2 and TMPRSS2, facilitating SARS-CoV-2 entry.	↑ H3K4me3	[10,28,29]
SLC6A20/SIT1	Positively regulated by ACE2, SLC6A20/SIT1 is suspected to reciprocally interact with ACE2 and enhance its activity.	↓ H3K27me3	[10,30,31]
IL-6	Present in high levels in the serum of COVID-19 patients, IL-6 is speculated to enhance viral entry by activating the AT1R signaling cascade.	↓ H3K27me3	[4,23,32,33]

NRP1 and NRP2, AT1R, CTSD and CTSL, several PGE2 receptors and SLC6A20 are the most well-studied mediators with potential implications in SARS-CoV-2 entry. The major common link among these large polypeptide molecules is that they are all induced by *mdig*, and hence, the worse prognosis of COVID-19 in smokers who are theoretically expected to carry high levels of these pro-entry mediators in their circulation [10]. IL-6 is perhaps the only pro-entry mediator not influenced by *mdig* in any known way.

## NRP1 & NRP2, CTSD & CTSL & AT1R contribute to SARS-CoV-2 infection in smokers

AT1R is supposedly one of the pillars of ACE2-driven SARS-CoV-2 entry, as the internalization of the S1 subunit of the spike protein through clathrin-mediated endocytosis requires the expression of AT1R, which is speculated to participate in the formation of a complex comprising ACE2 and S1. As a result, upregulation of AT1R might enhance S1 internalization and promote the infectivity of SARS-CoV-2 [22].

After binding ACE2, the S protein of SARS-CoV-2 is cleaved by the action of a protease named furin into S1 and S2 subunits, the latter of which is responsible for proper internalization of the virus. A transmembrane protein receptor found mainly in neurons, as well as other cell types, NRP1 was recently reported to bind the S1 subunit, and thus facilitate the separation of S2 from S1, resulting in increased infectivity of SARS-CoV-2 [34]. Similar to its counterpart, NRP2 can also bind the S1 subunit [21]. Both NRP1 and NRP2 were extracted in high concentrations from the lung tissue of deceased COVID-19 patients [35]. In addition to being induced by *mdig*, NRP1 and NRP2 can also be upregulated after exposure to arsenic [10]. As a toxic compound, arsenic was extracted in several different forms from the mainstream cigarette smoke condensate of four commercially available brands, with the most dominant forms being arsenate (As^5+^) and arsenite (As^3+^), both of which are considerably toxic [36].

A predictor of poor clinical outcome in COVID-19 patients [37], CTSD is significantly affected by *mdig* knockout because *mdig*^-^ cells exhibit decreased transcription of CTSD [10]. Unlike the other mediators, CTSD promotes SARS-CoV-2 entry through a rather complex pathway comprising furin and OPN. Furin is positively regulated by OPN [24], which is elevated in the serum of COVID-19 patients in its full length (FL-OPN) [25]. It is believed that cleaved forms of OPN might have higher activities than FL-OPN. As an enzyme, CTSD cleaves OPN at several sites, giving rise to fractionated OPN, which is relatively more active than FL-OPN [26]. In the case of COVID-19, there might be a hypothetical CTSD–OPN–Furin axis facilitating SARS-CoV-2 entry by accelerating the interaction between furin and the S protein.

## Smoking upregulates major glycosylation pathway genes via mdig

The SARS-CoV-2 spike protein is trimeric – that is, it is made up of three protomers (not to be confused with promoter), with each protomer containing 22 glycosylation sites. For this reason, glycosylation is of particular importance in the replication of SARS-CoV-2 because it can shield certain viral epitopes from the host immune response [38].

Silencing of *mdig* affects the expression of major genes involved in the glycolysation pathway by enhancing the occurrence of repressive histone marks such as H3K9me3 and H3K27me3 in these genes. *PIGN*, *HAS1* and *HAS2* are glycosylation-related genes, which happen to be downregulated in *mdig*^-^ lung epithelial cells [10]. PIGN was recently suggested to be a risk factor for severe COVID-19 [39]. As for the hyaluronan synthases, it is known that HAS1 and HAS2 are, respectively, upregulated following SARS-CoV-2 infection in mice [40] and humans, particularly in subjects with a more critical disease [41].

PIGN is a phosphoethanolamine transferase that mediates the biosynthesis of glycosylphosphatidylinositol (GPI)-anchor, a glycolipid responsible for the maintenance of cellular structure via anchoring proteins to the outer surface of the cell membrane. Impaired biosynthesis of GPI-anchor in neonates is associated with congenital disorders of glycosylation [42]. HAS1 and HAS2, on the other hand, are two of the three major hyaluronan synthases (HAS1-3) that catalyze the synthesis of hyaluronan from a precursor molecule known as uridine diphosphate N-acetylglucosamine (UDP-GlcNAc) [43], which is repressed in *mdig*^-^ cells itself. The synthesis-limiting effect of *mdig* knockout on UDP-GlcNAc is mediated through downregulation of UDP-GlcNAc pyrophosphorylase (UAP) and PGM2L1, which are two key enzymes in the synthesis of UDP-GlcNAc; the founding molecule for O-GlcNAcylaction [10].

Glycosylation is a major post-translational modification and is involved in the pathogenesis of SARS-CoV-2, particularly the viral entry phase. Considering the low transcription of glycosylation-associated genes in *mdig*^-^ lung epithelial cells, smoking might reinforce the expression of these genes in the respiratory tract, leading to enhanced glycosylation of the S protein and dampened immune response [10]. Strangely, however, smoking may also function as a protective factor in this case and counteract the infectivity of SARS-CoV-2 through a closely related, but discrete, pathway.

EZH2, a methyltransferase mediating the formation of H3K27me3, was recently suggested to be stabilized and functionally enhanced following its O-GlcNAcylation [18]. Hence, it is likely that an increased rate of O-GlcNAcylation, as a result of smoking [10], might be associated with promoter-specific enrichment of H3K27me3 in several COVID-19-related genes, especially *ACE2* [19]. Because EZH2 serves as the primary enzyme catalyzing the trimethylation of H3 at lysine residue 27, enhanced stability of this enzyme may promote H3K27me3 enrichment at the promoter of *ACE2*, interfering with SARS-CoV-2 entry. Nonetheless, this bizarre protective effect is most probably off set by the deleterious effects of arsenic, which is present in cigarette smoke [36]. Aside from the induction of *mdig* in lung epithelial cells [44], arsenic can also regulate histone modifications, such as H3K27me3, by triggering the phosphorylation of EZH2 at serine residue 21, which results in the impairment of EZH2 methyltransferase activity – and thus downregulation of H3K27me3 [45]. In this sense, the ultimate effect of arsenic is suspected to aggravate the overall transcription of *ACE2*.

## Smoking mediates epithelial–mesenchymal transition via EZH2

Smoking is associated with a near threefold increased transcription of EZH2 in human bronchial epithelial cells [46]. The EZH2-induced enhancement of H3K27me3 in the E-cadherin gene promoter reported in lung epithelial cells following exposure to cigarette smoke extract [47] is a good example of blood–air barrier-wise interplay between smoking and epigenetic regulation of gene transcription in the pulmonary system.

Consisting of alveolar epithelial and endothelial cells, along with the extracellular matrix lying in between, the blood–air barrier or alveolar–capillary barrier is a highly functional regulatory membrane-bound separator responsible for the alveolar exchange of respiratory gases (i.e., O_2_ and CO_2_). The alveolar epithelial cells lining the outermost surface of this barrier are tightly connected to one another by means of intercellular protein junctions, such as E-cadherin, the loss of which prompts epithelial-mesenchymal transition (EMT) in certain pathologic conditions, particularly malignancies. Although it was previously speculated that SARS-CoV-2, regardless of smoking history, might induce EMT at the blood–air barrier through the induction of TGF-β [17], it appears that enrichment of H3K27me3 at the promoter of E-cadherin might further accelerate an already progressing EMT process in the lungs of smokers.

## Smoking instigates inflammation in COVID-19 by inducing IL-6, IL-17 & PGE2

Expression of the viral S protein in lung epithelial cells infected with SARS-CoV-2 initiates a pro-inflammatory cascade characterized by increased levels of circulating IL-6 and soluble IL-6 receptor. The stimulatory effect of SARS-CoV-2 infection on IL-6 trans-signaling is mediated through the activation of AT1R [32], which in turn might aggravate viral entry [23].

In 2017, an investigation on a murine model of seizure suggested an epigenetic regulatory role for hyperthermia in the expression of IL-6. According to this study, hyperthermia might trigger the expression of IL-6 through downregulation of H3K27me3 in its promoter. The negative impact of hyperthermia on H3K27me3 enrichment is similar to the effect of EZH2 knockdown, which was shown to promote the active transcription of IL-6 by de-repressing or counteracting the effect of H3K27me3 [33].

On a more hypothetical ground, smoking could induce an airway-specific type of hyperthermia because it is essentially the act of inhaling hot smoke into the lungs. In line with this hypothesis, smoking can increase the temperature of the respiratory tract by an average of 1.25°C. This was determined by measuring the 5-minute post-smoking exhaled breath temperature [48], a variable with 98% specificity for predicting future development of COPD in smokers [4]. It is, however, unclear whether this rise of 1.25°C in temperature might be sufficient to induce promoter-specific H3K27me3 enrichment in certain genes, such as IL-6, in the epithelial cells of the respiratory tract. Regardless, there is still precedent for a connection between IL-6 and body heat because IL-6 is known to modulate core body temperature and induce fever, a systemic form of hyperthermia [49]. According to a systematic review, high serum levels of IL-6, presence of fever and current smoking are associated with at least 6, 9 and 12% of increased likelihood for development of severe COVID-19, respectively [50].

In addition to IL-6, the inflammatory phase of COVID-19 is potentially exacerbated under the influence of another distinguished pro-inflammatory cytokine, IL-17. *Mdig* knockout was shown to result in enhancement of H3K9me3 and H3K9me27 at the *IL17RD* gene, suggesting a positive regulatory effect for *mdig* on IL-17. In terms of immune response, SARS-CoV-2 infection is believed to induce the IL-17 signaling pathway, particularly in conditions associated with enhanced transcription of ACE2. This was confirmed by supplementation of lung epithelial cells with ACE2, which led to upregulation of certain transcripts shared with the IL-17 signaling pathway [51]. In fact, IL-17 was reported to be correlated with the severity of COVID-19 – that is, increased levels of IL-17 in COVID-19 patients may lead to a more severe disease [52]. A pro-inflammatory cytokine produced by a subfamily of T helper cells known as T helper 17 (Th17) cells [53], IL-17 is upregulated upon treatment with vitamin C, which can downregulate H3K9me3 in its promoter, and thus de-repress the transcription of IL-17 [54]. This is discussed later in the article.

In addition to IL-6 and IL-17, PGE2, a metabolite of arachidonic acid, is also thought to be implicated in the SARS-CoV-2-induced pulmonary inflammation observed in smokers. The role of PGE2, which predicts a worse prognosis for COVID-19 [28], is mostly established through its receptors – namely, PTGER2, PTGER3 and PTGER4. As such, *mdig*^-^ lung epithelial cells were found to have diminished expression of these three PGE2 receptors, a transcriptional change attained with downregulation of promoter-specific H3K4me3 marks. Thus, it would be sensible to assume that smoking maintains the transcription of PGE2 receptors by inducing *mdig* [10].

On the basis of an *in vitro* study, PGE2 upregulates ACE2 and TMPRSS2 in human gingival fibroblasts [29], an outcome that should also be appraised in the case of lung epithelial cells given that an increased concentration of PGE2 was found in the urine samples of COVID-19 patients, indicating generally higher levels of this cytokine in their circulation [55]. The disease-associated upregulation of PGE2 in COVID-19 happens to explain the seemingly contradictory findings of an investigation published in 2020, reporting lower levels of PGE2 in the BALF of mice exposed to cigarette smoke. Although exposure to cigarette smoke did in fact result in downregulation of PGE2 to as low as 100 pg/ml in the BALF of these mice [56], COVID-19 patients were found to have a minimum serum concentration of 1300 pg/ml for PGE2 [28], indicating the presence of PGE2 in even larger amounts at the primary site of infection, the respiratory tract.

In contrast to the inverse correlation between exposure to cigarette smoke and BALF-specific concentration of PGE2, there is a direct relationship between smoking and the number of macrophages mediating immune response at the pulmonary alveoli because a higher number of alveolar macrophages were found in the BALF of mice exposed to cigarette smoke. Taken together, smoking could exaggerate the pulmonary immune response by positively regulating the number of alveolar macrophages [56], while inducing the expression of PGE2 receptors in lung epithelial cells by means of *mdig* [10].

## The unfavorable effect of vitamin C on IL-17 is predominant in smokers

A 2010 investigation suggested that individuals with a history of cigarette smoking had lower serum levels of vitamin C compared with those without such history [57]. The adverse effect of smoking on the physiological interaction between vitamin C and the immune system, particularly white blood cells, was later confirmed by a study that reported decreased concentrations of leukocyte vitamin C in newly diagnosed tuberculosis patients with a history of smoking compared with nonsmokers [58].

The significance of vitamin C in the case of COVID-19-predisposing smoking-associated epigenetic modifications is not solely restricted to the IL-17. Vitamin C was also shown to mediate demethylation of *FOXP3* enhancer, leading to enhancer hypomethylation, an epigenetic modification associated with increased transcription [54]. Considering the inhibitory effect of FOXP3 on RORγt, an isoform of RORγ regulating the expression of IL-17 [53], vitamin C-induced upregulation of *FOXP3* is thought to repress IL-17 transcription and alleviate inflammation. This antiinflammatory effect can be explained by the defining role of *FOXP3* in differentiation of Tregs, which dampen inflammation and autoimmunity [59]. This speculation is consistent with the findings of a recent study reporting decreased numbers of Tregs in cigarette smokers [60]. It is interesting to note that the differentiation of Tregs is subject to regulation by several histone modifications, particularly H3K4me3, which is enriched at the promoter of *FOXP3* through the action of a methyltransferase known as SMYD3 [61], although it is not clear whether SMYD3 could be involved in the pathogenesis of COVID-19.

Thus, although IL-17 is positively regulated by the negative effect of vitamin C on its promoter-specific H3K9me3 marks, it can also be downregulated as a consequence of vitamin C-mediated FOXP3 enhancer hypomethylation. Accordingly, depending on the condition, insufficient levels of vitamin C might weaken its stimulatory and inhibitory effects on IL-7. The former effect appears to be more profoundly impaired in smokers, however, because they usually develop a more severe form of COVID-19-related inflammation [62].

## Smoking promotes the interaction between SLC6A20 & ACE2

Recently identified as a putative causal gene modulating COVID-19 severity [30], SLC6A20 – also known as SIT1 – is a high-affinity luminal L-proline transporter [31] highly expressed in lung epithelial cells [63]. The correlation between SLC6A20 and poor clinical outcomes in COVID-19 patients is suspected to stem from its affinity for ACE2 because SLC6A20 was reported to interact with ACE2. Although the nature of the interaction between the two proteins has not been clearly stated, ACE2 might induce the expression of SLC6A20 and its localization at the cell membrane [31]. Epigenetically speaking, SLC6A20 is upregulated as a result of *mdig*-induced depletion of H3K27me3 at its promoter [1]. Therefore, exposure to cigarette smoke might de-repress the transcription of SLC6A20, which is also under the influence of the SARS-CoV-2 spike protein receptor, ACE2 [31].

## Conclusion

Although the majority of theories in the present commentary are hypothetical, it is not lost on the scientific community that smoking is a strong predictor of poor clinical outcomes in the case of respiratory tract infections, such as the recently emerged COVID-19. The respective stimulatory and inhibitory effects of cigarette smoke on the enrichment of promoter or enhancer-specific active and repressive histone modifications associated with increased SARS-CoV-2 infectivity indicate that smoking is most certainly not an irrelevant comorbidity in the context of COVID-19. A significant number of receptors or mediators involved in the entry of SARS-CoV-2 into the lung epithelial cells and the glycosylation of its spike protein are known to be upregulated as a result of smoking-induced overexpression of *mdig*. Although the other pro-entry or pro-inflammatory mechanisms mentioned in this article are aptly supported by the current literature, one must not forget that every hypothesis needs to be confirmed by a well-structured investigation.

## Future perspective

The recently published evidence regarding the hazardous effects of the environmentally induced *mdig* on alveolar epithelial cells, among other cell types, which are mediated, to a great extent, by downregulation of repressive histone marks has significantly improved our understanding of the epigenetic background of occupational or environmentally triggered respiratory diseases. The findings regarding the crosstalk between these *mdig*-induced epigenetic alterations and increased vulnerability to the adverse outcomes of COVID-19, mentioned in the present article, might make a small contribution to organizing future clinical investigations in a way to better address for the epigenetic basis of pathogen–environment interactions.

**Executive summary**

- By inducing *mdig*, smoking might antagonize repressive histone marks that regulate the expression of certain key genes involved in the pathogenesis of COVID-19, particularly at the entry phase of SARS-CoV-2.
- Cigarette smoke contains several arsenic compounds that may induce the expression of *mdig*.
- Upon being triggered, *mdig* downregulates H3K9me3 and H3K27me3 at the promoters of ACE2, NRP1 and NRP2, AT1R, CTSD and CTSL, IL-6, SLC6A20 and PGE2 receptor 2-4, all of which are either known or speculated to favor infection with SARS-CoV-2 and the onset of COVID-19.
- Induction of *mdig* might also result in depletion of serum vitamin C levels, a common complication of smoking, and disinhibit the negative regulatory effect of vitamin C on IL-17, aggravating inflammation and leading to more severe COVID-19 infection in smokers.
